# Neighborhood-level socioeconomic factors moderate the association between physical activity and relative age effect: a cross-sectional survey study with Japanese adolescents

**DOI:** 10.1186/s12889-022-14052-5

**Published:** 2022-09-01

**Authors:** Takaaki Mori, Takumi Aoki, Kan Oishi, Tetsuo Harada, Chiaki Tanaka, Shigeho Tanaka, Hideki Tanaka, Kazuhiko Fukuda, Yasuko Kamikawa, Nobuhiro Tsuji, Keisuke Komura, Shohei Kokudo, Noriteru Morita, Kazuhiro Suzuki, Masashi Watanabe, Ryoji Kasanami, Taketaka Hara, Ryo Miyazaki, Takafumi Abe, Koji Yamatsu, Daisuke Kume, Hedenori Asai, Naofumi Yamamoto, Taishi Tsuji, Tomoki Nakaya, Kojiro Ishii

**Affiliations:** 1 Graduate School of Health and Sports Science, Doshisha University, Kyotanabe, Japan; 2 Faculty of Education, Miyagi Gakuin Women’s University, Sendai, Japan; 3 Education Unit, Humanities and Social Science Cluster, Research and Education Faculty, Kochi University, Kochi, Japan; 4 Department of Human Nutrition, Tokyo Kasei Gakuin University, Tokyo, Japan; 5 Faculty of Nutrition, Kagawa Nutrition University, Sakato, Japan; 6 Department of Medical Science and Technology, Faculty of Health Science, Hiroshima International University, Higashihiroshima, Japan; 7 Department of Psychology and Humanities, Faculty of Sociology, Edogawa University, Nagareyama, Japan; 8 Emeritus Professor, University of Toyama, Toyama, Japan; 9 Graduate School of Education, Shiga University, Otsu, Japan; 10 Faculty of Agriculture, Meijo University, Nagoya, Japan; 11 School of Health and Sports Sciences, Chukyo University, Toyota, Japan; 12 Department of Sports Cultural Studies, Hokkaido University of Education, Iwamizawa, Japan; 13 Faculty of Education, Ibaraki University, Mito, Japan; 14 Health and Sports Science Education, Faculty of Education, Nara University of Education, Nara, Japan; 15 Faculty of Education, Shimane University, Matsue, Japan; 16 Faculty of Human Sciences, Shimane University, Matsue, Japan; 17 Center for Community-Based Healthcare Research and Education (CoHRE), Head Office for Research and Academic Information, Shimane University, Izumo, Japan; 18 Faculty of Education, Saga University, Saga, Japan; 19 Faculty of Information Science and Technology, Osaka Institute Technology, Hirakata, Japan; 20 Faculty of Collaborative Regional Innovation, Ehime University, Matsuyama, Japan; 21 Faculty of Health and Sports Sciences, University of Tsukuba, Tokyo, Japan; 22 Graduate School of Environmental Studies, Tohoku University, Sendai, Japan; 23 Faculty of Health and Sports Science, Doshisha University, Kyotanabe, Japan

**Keywords:** Adolescents, Socioeconomic disadvantage, Relative age effect, Physical activity

## Abstract

**Background:**

Relative age effect is defined as a phenomenon where children born early generally perform better than children born later in the same cohort. Physical activity is an important factor that might be influenced by the relative age effect. Socioeconomic factors (e.g., parent’s income, education level) are also associated with the adolescent’s physical activity. However, no existing study has examined whether socioeconomic factors moderate the relative age effect on the adolescent’s physical activity. This study aims to clarify whether and how birth month and socioeconomic factors relate to organized sports and physical activity among adolescents in Japan.

**Methods:**

We conducted a questionnaire survey targeting 21,491 adolescents who live in a widespread neighborhood. We included 8102 adolescents (4087 males and 4015 females: mean age 13.1 ± 1.4) in the analysis. Based on the participants’ birth months, we divided them into four groups (April to June, July to September, October to December, January to March). We asked participants to report their organized sports participation. Using the International Physical Activity Questionnaire for Japanese Early Adolescents, we identified their moderate to vigorous physical activity (MVPA). Neighborhood-level socioeconomic factors (areal deprivation, average annual income, education level) were analyzed based on national surveys, such as the population census. We performed multilevel logistic and linear regression analysis for organized sports participation and MVPA, respectively. Moreover, a simple slope analysis was implemented if the interaction between birth month and socioeconomic factor was significant in the multilevel linear regression analysis.

**Results:**

Among males, relatively younger adolescents (adolescents who were born later in the same grade) were less likely to participate in organized sports activites (OR=0.90, 95% CI 0.82–0.97, p<0.05), while both males and females engaged in less MVPA (b=-0.54, b=-0.25, p< 0.01, respectively). We observed an interaction between birth month and socioeconomic factors. Among males in low-income neighborhoods, and females in more deprived neighborhoods, relatively younger adolescents engaged in less MVPA.

**Conclusions:**

Socioeconomic factors moderate the relative age effect on adolescents’ physical activity. The relative age effect on adolescents’ physical activity might be more likely to appear among adolescents from socioeconomically disadvantaged neighborhoods.

## Background

To date, many studies have shown that efficient physical activity improve various health indicators among children, such as physical fitness (e.g., physical strength, cardiopulmonary function), cardiovascular health (e.g., blood pressure, insulin secretory capacity), bone health (e.g., bone mineral density), cognitive function (e.g., memory, academic performance) and mental health (e.g., reduction of depression risk). Therefore, the World Health Organization recommends that children aged 5–17 years engage in an average of at least 60 min of moderate to vigorous intensity physical activity per day [1]. Additionally, some studies have suggested childhood physical activity and health status might influence physical activity and health status in adulthood. Thus, it is important to establish active habits (i.e., engage in physical activity) during childhood and to keep active until adulthood [2].

In Japan, children enroll in school in April; the school year begins on April 2 and ends on April 1 of the following year. Thus, children born between January 1 and April 1 are almost one year younger than those born at the beginning of the school year. Relative age effect is defined as gaps caused due to chronological age differences among students of the same grade [3]. This phenomenon presupposes that children born early in a cohort generally perform better in various situations (e.g., sports, academic examinations) than those born later in the same cohort. This may occur as children born between January 1 and April 1 may exhibit poorer physique and physical fitness. Many previous studies showed that those who were born later are likely to have poor scholastic ability and poor socio-emotional development [4, 5]. Ultimately, the relative age effect remains until adulthood. For example, individuals born later were found to be less likely to go to university at the age of 18 years [4]. Other previous studies have shown that the distribution of the birth month of athletes across different sports was unbalanced, indicating a higher proportion of athletes born earlier in the academic year than those born later [3, 6, 7]. Therefore, it is essential to understand the relative age effect and to strive to mitigate the disadvantages caused by the relative age effect from childhood.

A Japanese study reported that the relative age effect of physical fitness exists among general Japanese primary school students regardless of sex and age; thus, birth month and level of sports activity can be considered as factors that explain the inequality in physical fitness [8]. In contrast, another study showed that relatively younger adolescents, with better physical fitness than relatively older adolescents, are more likely to participate in physical activities. This indicates that physical activity might be an important factor that mitigates the relative age effect [9].

Socioeconomic status (SES), such as parents’ income and education level, is one of the factors that influences adolescents’ sports participation and physical activity [10–13]. Adolescents with low SES are less likely to participate in sports and to engage in physical activity than adolescents with high SES. Adolescents with low SES have difficulty participating in sports due to lack of various supports (e.g., financial supports, social supports from friends or parents) [14]. In addition, neighborhood socioeconomic factors are associated with healthy behavior; for example, socioeconomically disadvantaged neighborhoods tend to have insufficient recreational facilities and few opportunities for individuals to participate in sports [15–17]. However, no studies have yet examined whether there are neighborhood disparities in the relative age effect among adolescents. Previous studies on the relative age effect have only targeted one or a few areas and only considered differences within the areas. In response to this gap in the literature, we not only focused on differences within the area but also differences between plural areas based on socioeconomic factors. Next, we supposed two hypotheses. First, we wagered that both birth month and socioeconomic factors are directly associated with sports participation and physical activity among adolescents; specifically, we reasoned that the later an adolescent’s birth month and the more socioeconomically disadvantaged the neighborhood, the lower their rates of sports participation and physical activity were likely to be. Second, proposed that the relative age effect of physical activity is more strongly influenced by birth month in socioeconomically disadvantaged neighborhoods, where the relative age effect can be seen more clearly. Notably, there are few racial and ethnic minorities in Japan and economic disparity in the nation has widened with the increase in poverty [18]. In sum, this study’s purpose was to clarify how birth month and socioeconomic factors are related to the organized sports participation and physical activity (which represent “exercise” in this study) of adolescents across Japan.

## Methods

### Target area and sample

Japan mainly consists of eight regions (Hokkaido, Tohoku, Kanto, Chubu, Kinki, Chugoku, Shikoku, and Kyushu). The Ministry of Internal Affairs and Communications of Japan determines city scale based on city population and classifies cities into the following categories: large cities (population of more than 500 thousand), core cities (population of 200–500 thousand or prefectural capital), medium cities (population of 100–200 thousand), small cities (population of 10–100 thousand), and town and village (population of less than 10 thousand) [19].

**Fig. 1:**
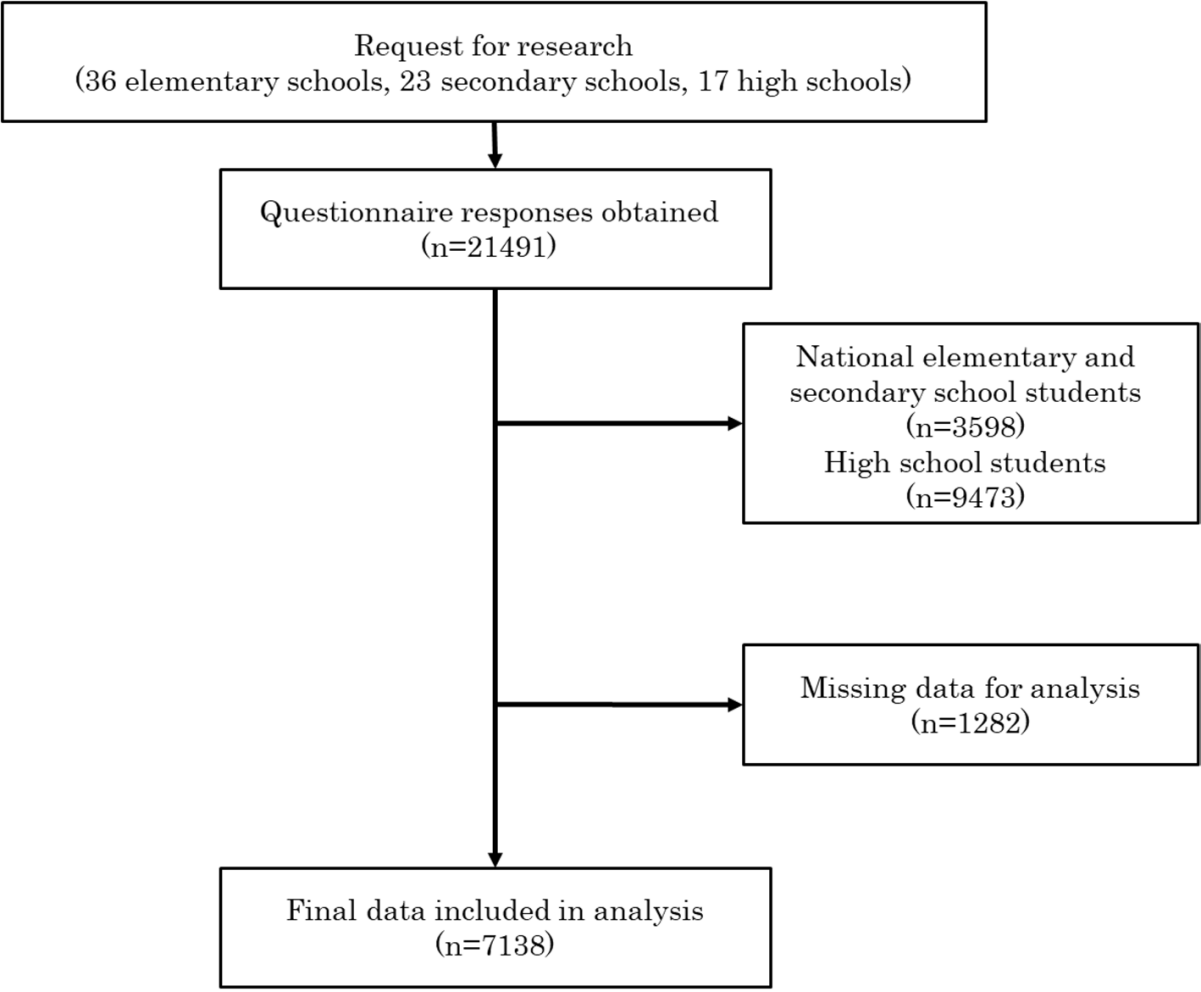
Protocol for recruiting research participants for analysis

Our research team comprised 18 researchers from 15 research institutions. We selected and mailed surveys to 78 schools from the eight regions and asked them to complete them. Among them, 76 schools (61 public school, 12 national school, 3 private school) of 78 schools accepted the survey. [20] The survey involved a self-report questionnaire. We asked the schools to give it to 11- to 18-year-old adolescents between 2017 and 2019. In sum, we obtained 21491 questionnaire responses. The purpose, method, benefits, and risks of this study were explained to the principals of the schools. We also explained to the participants that their private information would be protected and that answers to the questionnaires were not related to their school records. Participants provided consent before answering the questionnaire. We excluded 3598 national primary school and secondary school students and 9473 high school students from this study because it was difficult to specify their school district. In Japan, national schools and high schools do not have school districts, and some students may go to school far from their homes. Since we could not specify these participants' addresses, we could not assess their neighborhood environments. We also excluded 318 adolescents due to missing data regarding sex. As a result, 8102 adolescents (4087 males and 4015 females from 48 schools, 11–15 years old) were included in the analysis (Fig. 1).

### Individual-level characteristics

We obtained basic information regarding each participant’s sex, birth year, birth month, height, body weight, and organized sports participation through their self-reports. We divided birth month into four groups: Q1 (April to June), Q2 (July to September), Q3 (October to December), and Q4 (January to March). We calculated the body mass index percentile by sex and age from the height and body weight. Those with a body mass index above the 85^th^ percentile were categorized as overweight [21]. We asked participants to report their organized sports participation. Those who participated in least one organized sport (e.g., school sports club activity, neighborhood sports group activity, private sports lesson) were categorized as “Active,” and those who did not participate in any sports activities were categorized as “Inactive.”

We used the International Physical Activity Questionnaire for Japanese Early Adolescents to assess students’ physical activity levels. We also asked students, “How often do you engage in physical activity per week?” and “How long do you engage in physical activity per day on average?”, regarding moderate physical activity (MPA) and vigorous physical activity (VPA) [22]. Based on a previous study, moderate to vigorous physical activity (MVPA) per day was calculated as follows [23].

$$MVPA = \left\{(MPA frequency \times MPA duration) + (VPA frequency \times VPA duration)\right\} / (7 days)$$

### Neighborhood-level characteristics

Each board of education in Japan determines the school district based on geographical conditions (e.g., streets and rivers), neighborhood traditions, and residents’ preferences [24]. We defined a school district as a neighborhood unit in this study because the size of the school district corresponded to the daily living area [25, 26]. Public school students in Japan must go to their designated school as per their residential address, and they are instructed not to go out of their school district without their guardians [27].

**Fig. 2:**
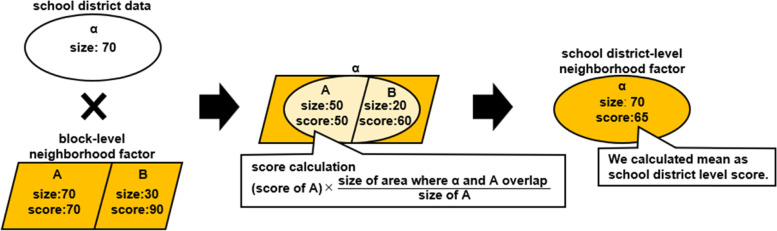
The procedure of areal weighting interpolation in this study

To apply the results of the national study data collected by the municipalities or by the block (*cho-cho-aza*), we conducted weighting interpolation with a geographic information system (Fig. 2). First, we overlapped a block-level neighborhood factor and school district polygon data. Then, we computed scores by the ratio of the size of the overlapped area per size of each school district. We calculated the mean of the overlapped area in the school district as the school district level score.

In Japanese culture, asking someone’s academic background or income is frowned upon. A previous study in Japan also reported that only a few participants reported their academic background and income [28]. Thus, we substituted three neighborhood socioeconomic factors: areal deprivation, neighborhood education level, and average annual income. Areal deprivation is an index that reflects the relative size of poor household ratio. We used the Areal Deprivation Index (ADI), a weighted index wherein the following eight variables were associated with poverty from the Population Census in 2010: proportion of elderly single households, elderly couple households, single mother households, rental housing households, sales and service workers, agricultural workers, blue-collar workers, and unemployed persons [29–31]. We overlapped block-level ADI and school district data and calculated the mean of each block that was composed in a school district as neighborhood ADI. In addition, we obtained data regarding income and estimated the average annual income from the Housing and Land Survey in 2013 and the Population Census in 2015 [32, 33]. The division of the population according to income consisted of six classes: less than 3 million yen, 3–5 million yen, 5–7 million yen, 7–10 million yen, 10–15 million yen, and more than 15 million yen. We multiplied the class value by the number of households in each income class, summed up the product, and divided it by the number of general households in the school districts. We estimated block-level income data by overlapping the municipality-level income data obtained from the Housing and Land Survey in 2013 and block-level population data from the Population Census in 2015. Then, we overlapped block-level income data and school district data and calculated neighborhood-level income data. Furthermore, we calculated the proportion of people who graduated from university or graduate school from the Population Census in 2015 to yield neighborhood education levels. Finally, we referred to population data from the Population Census in 2015 [32] and calculated the population density of school districts.

### Statistical analysis

We estimated lacking data by multiple imputation (the frequency of multiple imputation was five) and calculated the average imputed score for the analysis. In addition, MVPA was normalized by a Box-Cox transformation.

Since this study included both individual-level and neighborhood-level variables, we used multilevel modeling. First, we examined only the birth month (Model 1) to calculate the interclass correlation coefficient. Then, we added each socioeconomic factor to Model 1 (Model 2; Model 2a: Areal deprivation; Model 2b: Average annual income; Model 2c: neighborhood education level). Furthermore, we examined the cross-level interaction between birth month and each socioeconomic factor (Model 3). As for Model 3, birth month was included as a random effect. To prepare for multilevel regression analysis, we used the centering method for all independent variables and covariates.

We conducted multilevel logistic regression analysis to examine whether adolescent organized sports participation was associated with birth month and socioeconomic factors. We estimated a 95% confidence interval (95% CI) using the Wald test. We also ran multilevel linear regression analysis to clarify whether adolescent MVPA was related to birth month and socioeconomic factors. If statistical significance was observed, we conducted a simple effect analysis [34]. To express cross-level interaction, we estimated a single slope of the birth month at mean ± 1 standard deviation [35].

Males are more likely to participate in sports and engage in physical activity more frequently than females [36, 37]. Additionally, males are more likely to demonstrate the relative age effect than females [38]. Therefore, all models considered sex. We adjusted for the following covariates: age, body weight, and population density. We conducted statistical analysis using SPSS 28.0 and EZR (Easy R) [39], and statistical significance was set at *p* < 0.05.

## Results

Table 1 shows the individual-level characteristics of the study participants. The mean age was almost the same for males and females, and few adolescents were overweight. Regardless of sex, the distribution of age at birth was unbiased. Overall, 77.8% males and 52.5% females participated in organized sports. Average MVPA time was 77.6 ± 68.9 min/day for males and 55.4 ± 69.9 min/day for females. Distribution of MVPA was skewed for both males (median: 60.0 min/day, first quartile: 22.9, third quartile: 115.7) and females (median 34.3 min/day, first quartile: 8.6, third quartile: 85.7). After normalizing MVPA by a Box-Cox transformation, the average of MVPA time was 10.1 ± 5.6 min/day for males and 5.4 ± 3.5 min /day for females. Table 2 shows the neighborhood-level characteristics of this research.

**Table 1:** The individual-level characteristics of current research participants

	Male (*n* = 4088)		Female (*n* = 4015)	
	Mean (SD) or n (%)		Mean (SD) or n (%)	
Individual characteristics				
Age (years)	13.1	( 1.4)	13.1	( 1.4)
Height (cm)	154.3	( 11.9)	151.1	( 7.9)
Body weight (kg)	44.9	( 11.4)	42.5	( 8.3)
Body mass index percentile	41.3	( 28.1)	38.5	( 27.1)
Weight status				
Overweight	419	( 10.2%)	229	( 6.8%)
Non-Overweight	3668	(89.8%)	3136	(93.2%)
Birth month				
Q1(April-June)	1025	(25.1%)	830	(24.7%)
Q2(July–September)	1129	(27.6%)	914	(27.2%)
Q3(Octorber-December)	933	(22.8%)	792	(23.5%)
Q4(January-March)	1000	(24.5%)	829	(24.6%)
Organized sports participation				
Joining	3180	(77.8%)	1803	(53.6%)
Not-Joining	907	(22.2%)	1562	(46.4%)
MVPA time (min/day)	77.6	( 68.9)	55.2	( 60.4)

Estimated mean, SD and number (Lacking data was estimated by multiple imputation.)

*SD* Standard deviation, *MVPA* Moderate to vigorous physical activity

**Table 2:** The neighborhood-level characteristics of the participating schools in this study (48 schools)

	Mean	SD
Areal deprivation	42.4	25.8
Higher education level(%)	15.1	6.8
Average annual income(× 10^4^ yen)	548.1	50.6
Population density(/ha)	12.7	19.3

*SD* Standard deviation

Tables 3 and 4 show the results of multilevel logistic regression analysis: birth month was associated with organized sports participation only for male (Odds Ratio [OR] = 0.90, 95% CI 0.82–0.97, *p* < 0.01, Model 1, Table 3). However, birth month was not significantly associated with organized sports participation among females. None of the socioeconomic factors were associated with adolescent organized sports participation (Model 2).

**Table 3:** The neighborhood-level characteristics of the participating schools in this study (48 schools)

	Model 1 OR(95%CI)	Model 2a OR(95%CI)	Model 2b OR(95%CI)	Model 2c OR(95%CI)
Fixed effects				
Intercept	3.08 (2.31–4.10)**	3.08 (2.30–4.12)**	3.13 (2.29–4.28)**	3.11 (2.32–4.16)**
Birth month	0.90 (0.82–0.97)*	0.90 (0.23–0.97)*	0.90 (0.83–0.98)*	0.90 (0.82–0.98)*
Socioeconomic factor				
Areal deprivation		1.00 (0.99–1.01)		
Average annual income			1.00 (0.99–1.01)	
Education level				1.01 (0.96–1.06)

*OR* odds ratio, *95%CI* 95% confidence interval

Model 1: Only birth month was considered. No socioeconomic factor was added

Model 2: Birth month and one socioeconomic factor(Model 2a: Areal deprivation, Model 2b: Average annual income, Model 2c: Education level)

All models were adjusted for age, body weight, population density

^*^p < 0.05, **p < 0.01, Interclass correlation coefficients = 0.14, Design effect = 12.72

**Table 4:** Estimates from multilevel logistic modeling for female adolescents’ organized sports participation (*n* = 4015)

	Model 1	Model 2a	Model 2b	Model 2c
	OR(95%CI)	OR(95%CI)	OR(95%CI)	OR(95%CI)
Fixed effects				
Intercept	0.94 (0.75–1.18)	0.94 (0.74–1.19)	0.94 (0.74–1.18)	0.92 (0.73–1.17)
Birth month	0.94 (0.87–1.00)	0.94 (0.87–1.00)	0.94 (0.87–1.00)	0.93 (0.87–1.00)
Socioeconomic factor				
Areal deprivation		1.00 (0.99–1.01)		
Average annual income			1.00 (1.00–1.00)	
Education level				0.98 (0.95–1.02)

OR: odds ratio, 95%CI: 95% confidence interval

Model 1: Only birth month was considered. No socioeconomic factor was added

Model 2: Birth month and one socioeconomic factor(Model 2a: Areal deprivation, Model 2b: Average annual income, Model 2c: Education level)

All models were adjusted for age, body weight, population density

Interclass correlation coefficients = 0.09, Design effect = 8.48

**Fig. 3:**
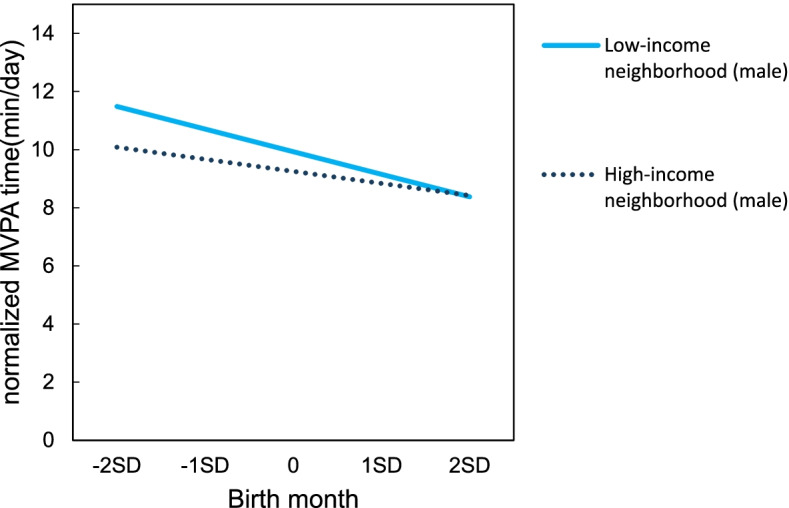
Simple slope of relative age effect by average annual income for males

**Fig. 4:**
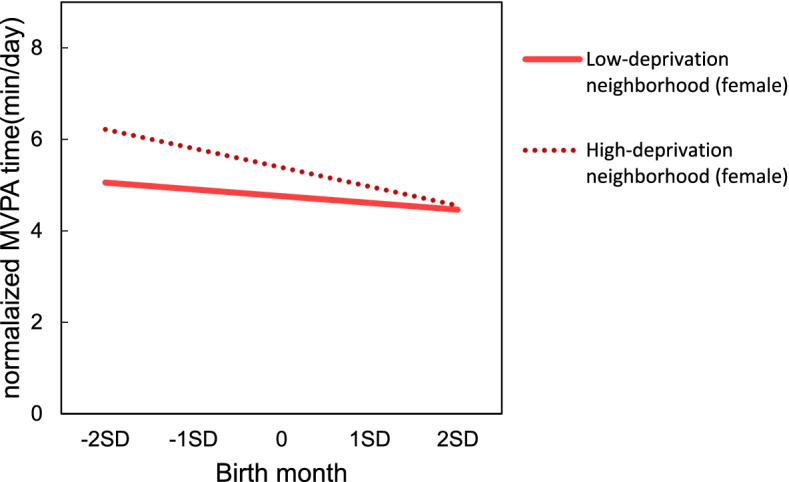
Simple slope of relative age effect by areal deprivation for females

Tables 5 and 6 show the estimated adolescent MVPA from multilevel linear modeling. Relatively younger adolescents reported negative associations with MVPA for both males (b = -0.54, *p* < 0.01) and females (b = -0.25, *p* < 0.01). No association was found between socioeconomic factors and adolescent MVPA. Furthermore, we used cross-level interaction modeling in order to examine whether neighborhood-level socioeconomic factors moderated the association between birth month and adolescent MVPA. We observed a significant cross-level interaction between birth month and average annual income among males (b = 0.002, *p* < 0.05, Table 5). Relatively younger adolescents in low-income neighborhoods reported associations with less MVPA time (males: b = -0.70, *p* < 0.01, Fig. 3, compared to those in high-income neighborhoods. Further, there was a significant interaction between birth month and areal deprivation among females (b = -0.004, *p* < 0.05, Table 6). Relatively younger females in more deprived areas were likely to engage in less MVPA (b = -0.37, *p* < 0.01, Fig. 4); no significant association was observed in less deprived areas. No interaction was found between birth month and education level for MVPA among both males and females.

**Table 5:** Estimates from multilevel linear modeling for male adolescents’ Moderate-to-Vigorous Physical Activity (MVPA) (*n* = 4087)

	Model 1	Model 2a	Model 2b	Model 2c	Model 3a	Model 3b	Model 3c
	b(SE)	b(SE)	b(SE)	b(SE)	b(SE)	b(SE)	b(SE)
Fixed effects							
Intercept	9.62 (0.38)**	9.56 (0.39)**	9.57 (0.39)**	9.63 (0.40)**	9.57 (0.39)**	9.58 (0.39) **	9.64 (0.40)**
Birth month	-0.54 (0.08) **	-0.55 (0.08)**	-0.56 (0.08)**	-0.55 (0.08) **	-0.55 (0.08)**	-0.55 (0.08) **	-0.55 (0.09)**
Socioeconomic factor							
Areal deprivation		0.01(0.01)			0.01 (0.01)		
Average annual income			-0.01(0.01)			-0.01 (0.01)	
Education level				0.02 (0.06)			0.02(0.06)
Cross-level interaction							
Birth month*Areal Deprivation					0.00(0.00)		
Birth month*Average annual income						0.00 (0.00)*	
Birth month*Education Level							0.02 (0.01)

MVPA was normalized by a Box-Cox transformation

*SE* Standard error

Model 1: Only birth month was considered. No socioeconomic factor was added

Model 2: Birth month and one socioeconomic factor(Model 2a: Areal deprivation, Model 2b: Average annual income, Model 2c: Education level)

Model 3: Birth month, one socioeconomic factor and one cross-level interaction (Model 3a: Birth month*Areal deprivation, Model 3b: Birth month*Average annual income, Model 3c: Birth month*Education level)

All models were adjusted for age, body weight, population density

^**^p < 0.01, *p < 0.05, Interclass correlation coefficients = 0.08, Design effect = 7.35

**Table 6:** Estimates from multilevel linear modeling for female adolescents’ Moderate-to-Vigorous Physical Activity (MVPA) (*n* = 4015)

	Model 1	Model 2a	Model 2b	Model 2c	Model 3a	Model 3b	Model 3c
	b(SE)	b(SE)	b(SE)	b(SE)	b(SE)	b(SE)	b(SE)
Fixed effects							
Intercept	5.09 (0.20)**	5.06 (0.20)**	5.07 (0.20)**	5.07 (0.20)**	5.06 (0.20)**	5.07 (0.20)**	5.07 (0.20)**
Birth month	-0.25 (0.05)**	-0.26 (0.06)**	-0.26(0.06)**	-0.25 (0.05)**	-0.26 (0.05)**	-0.25 (0.05)**	-0.26 (0.05)**
Socioeconomic factor							
Areal deprivation		0.01 (0.01)			0.01 (0.01)		
Average annual income			-0.00 (0.00)			-0.01 (0.00)	
Education level				-0.02 (0.03)			-0.02 (0.03)
Cross-level interaction							
Birth month*Areal Deprivation					-0.00 (0.00)*		
Birth month*Average annual income						0.00 (0.00)	
Birth month*Education level							0.01 (0.01)

MVPA was normalized by a Box-Cox transformation

*SE* Standard error

Model1: Only birth month was considered. No socioeconomic factor was added

Model2: Birth month and one socioeconomic factor(Model 2a: Areal deprivation, Model 2b:Average annual income, Model 2c: Education level)

Model3: Birth month, one socioeconomic factor and one cross-level interaction (Model 3a: Birth month*Areal deprivation, Model 3b: Birth month*Average annual income, Model 3c: Birth month*Education level)

All models were adjusted for age, body weight, sports club activities, population density

^**^p < 0.01, *p < 0.05, Interclass correlation coefficients = 0.05, Design effect = 4.74

## Discussion

This study showed that among relatively younger adolescents, males were less likely to participate in sports and that physical activity was not associated with birth month among females. Additionally, birth month was significantly associated with MVPA for both males and females, which shows that adolescents spend less time on MVPA. Moreover, we found significant interactions between birth month and average annual income among males and between birth month and areal deprivation among females. The simple slope analysis revealed that the relative age effect of physical activity may emerge in socioeconomically disadvantaged neighborhoods.

Previous studies have reported that relatively younger adolescents are likely to spend less time playing sports [28] and more likely to have poorer physical fitness [8, 9, 40] and lower noncognitive skills (e.g., self-efficacy, ability belief) [4, 5]. Considering these previous studies, relatively younger adolescents may be more likely to be reluctant to play sports. In addition, we only found a relative age effect for organized sports participation among males. Although the reason for this is not clear, there are some possible explanations. First, more females might participate in sports than females. In this study, the percentage of males who participate in organized sports is higher (77.8%) than the percentage of female (53.6%). Second, the kind of physical activity might influence these results. While many males are likely to naturally participate in organized sports (e.g., soccer, basketball, baseball), females are likely to enjoy playing with a small group (e.g., badminton, athletic play with playground equipment) [41]. Based on the above, males have more opportunities to participate in sports, which would facilitate clearer results for males. However, relatively younger males may be less likely to participate in sports because they may have fewer opportunities to play active roles in organized sports.

Socioeconomic factors were not directly associated with sports participation and physical activity. Previous studies have shown that adolescents demonstrate weaker association between socioeconomic factors and sports participation and physical activity duration than preschool children [13]. In Japan, most secondary school students belong to school activity clubs, which are generally scheduled after school. Although we could not specify what type of club activity adolescents belonged to, school sports club activities may weaken the influence of socioeconomic factors on organized sports participation and physical activity.

The relative age effect of physical activity might be more likely to emerge in socioeconomically disadvantaged neighborhoods. Although no study has examined how socioeconomic factors moderate the association between birth month and adolescent physical activity, there are some possible explanations. First, low SES adolescents are less likely to participate in sports because of financial instability [13, 14], and socioeconomically disadvantaged neighborhoods are less likely to have parks [16, 17] or sports facilities [42–44]. Youth in socioeconomically disadvantaged neighborhoods might have fewer opportunities to play active roles in various sports than youth in socioeconomically advantaged neighborhoods. Thus, youth in socioeconomically disadvantaged neighborhoods were more likely to cause relative age effect of physical activity. On the other hand, relatively younger adolescents who live in affluent neighborhoods might be able to more easily engage in alternative sports, which might mitigate the relative age effect of physical activity. Additionally, relatively older adolescents in socioeconomic disadvantaged neighborhoods engaged in the most MVPA in this study. This seemed to occur due to the Matthew effect, in which the superiors get superior, and the inferiors get inferior in particular situation [45]. In socioeconomically disadvantaged environments, relatively older adolescents might have more opportunities to play active roles in sports, which might be why they engage in more physical activity.

Thus, as mentioned above, physical activity is an important factor that mitigates the relative age effect [9]. However, since relatively younger adolescents might have undergone fewer successful experiences with sports than their relatively older counterparts, they may be more likely to become inactive. Therefore, teachers and coaches should encourage relatively younger adolescents to engage in physical activity. For example, teachers and coaches must formulate sports rules to enable equal active roles among adolescents. In addition, it is essential for schools and sports facilities to provide later-born adolescents with adequate opportunities to engage in physical activity and to play active roles in sports by participating in various sports. In this study, we observed a relative age effect of physical activity in both male and female adolescents from socioeconomically disadvantaged areas. Socioeconomically disadvantaged adolescents are less likely to engage in physical activity owing to their financial instability. Thus, schools and public sports facilities should support socioeconomically disadvantaged adolescents to engage in sports at minimum financial costs.

This study was the first to examine how socioeconomic factors moderate the association between birth month and adolescent physical activity. In addition, we defined school districts as neighborhood units, and applied municipality and block-level data for neighborhood-level data. Furthermore, we showed the relative age effect of adolescent organized sports participation and physical activity by adjusting various individual and neighborhood characteristics. Nevertheless, this study has some limitations. First, this was a cross-sectional study; therefore, we could not address causal relations. Second, while some previous studies examined physical fitness as a relative age effect outcome [8, 9, 40], we did not research participants’ physical fitness. However, previous studies have reported that high-level physical fitness is related to more MVPA time [9]. Thus, those who spent more time on MVPA seemed to have a higher level of physical fitness in this study. Third, since we could not ask participants about their SES, we could not address household SES levels. In Japan, cultural norms make it difficult to directly ask for parents’ SES. Therefore, we substituted neighborhood-level socioeconomic factors based on demographics, such as census data. Previous studies have reported that individual-level socioeconomic factors are more strongly related to adolescent physical activity than neighborhood-level factors [11, 12]. We might observe a clearer association between socioeconomic factors and adolescent physical activity upon utilizing individual-level socioeconomic factors.

## Conclusion

We uncovered the association between the relative age effect and adolescents’ organized sports participation and physical activity for both male and female adolescents across Japan. We observed a significant interaction between birth month and socioeconomic factors; the relative age effect of physical activity was seen only for adolescents who lived in socioeconomically disadvantaged neighborhoods. Since few studies have examined the relationship between the relative age effect and neighborhood type, further studies are required to address neighborhood characteristics where the relative age effect is likely to appear.

## Data Availability

The datasets analyzed during the current study are not publicly available because the publication of datasets that may identify schools is prohibited in Japan; however, the datasets are available from the corresponding author upon reasonable request.

## Abbreviations

SES: Socioeconomic status
MPA: Moderate physical activity
VPA: Vigorous physical activity
MVPA: Moderate to vigorous physical activity
ADI: Areal Deprivation Index
